# Liver Functions in Patients with Chronic Liver Disease and Liver Cirrhosis: Correlation of FLIS and LKER with PALBI Grade and APRI

**DOI:** 10.2174/0115734056388870250818114743

**Published:** 2025-09-18

**Authors:** Ahmet Cem Demirşah, Elif Gündoğdu

**Affiliations:** 1 Department of Radiology, Afyonkarahisar Bolvadin State Hospital, Afyonkarahisar, Turkey; 2 Department of Radiology, Eskişehir Osmangazi University, Faculty of Medicine, Eskişehir, Turkey

**Keywords:** Liver function tests, Liver diseases, Dynamic contrast-enhanced magnetic resonance imaging, Gadolinium ethoxybenzyl DTPA, End-stage liver disease, Magnetic resonance imaging

## Abstract

**Introduction:**

In chronic liver disease (CLD) and liver cirrhosis (LC), assessing hepatic function and disease severity is crucial for patient management. This study aimed to evaluate the relationship between platelet-albumin-bilirubin (PALBI) grade and aspartate aminotransferase/platelet ratio index (APRI) with the functional liver imaging score (FLIS) and liver-to-kidney enhancement ratio (LKER) using gadolinium ethoxybenzyl diethylenetriamine pentaacetic acid (Gd-EOB-DTPA)-enhanced hepatobiliary phase (HBP) magnetic resonance imaging (MRI).

**Methods:**

After applying exclusion criteria, 86 patients with CLD or LC who underwent Gd-EOB-DTPA-enhanced MRI between January 2018 and October 2023 were included. APRI and PALBI grades were calculated from laboratory data. FLIS was determined as the sum of three HBP imaging features (liver parenchymal enhancement, biliary excretion, and portal vein sign), with each scoring 0–2. LKER was calculated by dividing liver signal intensity by kidney intensity using region of interest (ROI) measurements. Spearman’s correlation was used to assess relationships between the variables.

**Results:**

APRI showed a weak negative correlation with both FLIS (r = –0.327, p = 0.02) and LKER (r = –0.308, p = 0.004). PALBI showed a moderate negative correlation with FLIS (r = –0.495, p = 0.001) and LKER (r = –0.554, p = 0.0001).

**Discussion:**

FLIS and LKER moderately correlated with PALBI and weakly with APRI. LKER may be a more practical tool due to its quantitative nature. Despite limitations, combining imaging and lab-based scores could enhance liver function assessment.

**Conclusion:**

FLIS and LKER can validate, rather than predict or exclude, liver dysfunction in CLD and LC.

## 1 INTRODUCTION

Chronic liver disease (CLD), which occurs due to various causes, such as chronic viral hepatitis (*e.g*., hepatitis B and C), alcoholic liver disease, nonalcoholic fatty liver disease (NAFLD), autoimmune hepatitis, and metabolic or genetic disorders (*e.g*., hemochromatosis, Wilson’s disease), is defined as a progressive deterioration of liver functions lasting for more than six months [1]. The process, which begins with inflammation in the early stages, progresses over time, and liver fibrosis is the most common outcome of chronic liver injury [2]. Eventually, progressive liver fibrosis can lead to liver cirrhosis (LC), the final stage of CLD. The liver performs different functions, such as synthesizing clotting factors and other proteins, detoxifying harmful metabolism products, and excreting bile [1]. In CLD and LC, determining the status of these functions and the severity of the disease is very important in patient management. For this reason, some scoring systems have been developed depending on various clinical and laboratory parameters indicating liver functions, such as albumin-bilirubin (ALBI) grade, platelet-albumin-bilirubin (PALBI) grade, Child-Turcotte-Pugh (CTP) score, aspartate aminotransferase/platelet ratio index (APRI), and the model of end-stage liver disease (MELD) [3].

Gadolinium ethoxy benzyl diethylenetriamine pentaacetic acid (Gd-EOB-DTPA; gadoxetic acid disodium) is a liver-specific contrast agent for magnetic resonance imaging (MRI). Approximately 50% of injected Gd-EOB-DTPA is taken into functioning hepatocytes by organic anion-transporting polypeptides (OATPs) located in the liver membrane and then excreted into the biliary tract by multidrug resistance-associated protein 2 [4, 5]. Gd-EOB-DTPA reflects anatomical information regarding the biliary tract, blood supply, and liver function. Since it is related to liver function, it can be used for evaluating a patient’s liver function in LC and predicting liver failure after liver tumor resection [6]. Bastati *et al*. developed a functional liver imaging score (FLIS) derived from Gd-EOB-DTPA-enhanced HBP MRI [7]. The three parameters included in the FLIS are based on a visual scoring assessment of liver parenchymal enhancement (EnQS, 0-2), biliary contrast excretion (ExQS, 0-2), and signal intensity of the portal vein relative to the liver parenchyma, that is, the portal vein sign (PVsQS, 0-2). The FLIS is calculated as the sum score of these three parameters [7]. Bastati *et al*. demonstrated that the FLIS derived from gadoxetic acid–enhanced MRI can identify patients with advanced chronic liver disease who are at increased risk for a first hepatic decompensation and increased mortality [8]. Various studies have shown moderate and high correlations between the quantitative Gd-EOB-DTPA-enhanced hepatobiliary phase (HBP) MRI scores and liver function parameters [3, 9]. Ozgul D *et al*. found FLIS obtained from GA-enhanced MRI in the hepatobiliary phase to moderately correlate with CTP classification and ALBI grade in HCC patients, with excellent inter-reader agreement [10]. Aslan *et al*. showed excellent agreement with FLIS among readers with different levels of experience, and they claimed that it can be used with high accuracy and reproducibility regardless of experience [3]. Similarly, in a meta-analysis, it was shown that FLIS and its three subcategories displayed almost perfect inter-reader reliability [11]. Despite these high agreements, the relationship between liver functions and FLIS scores has shown variations in different studies. Moderate, good, and excellent agreements have been reported in various studies [3, 12, 13].

Although GD-EOB-DTPA is a hepatobiliary-specific agent, it also possesses the property of an extracellular contrast agent, with excretion being 50%-50% in individuals with normal liver and kidney function [13]. However, renal excretion increases in individuals with liver dysfunction. In patients with CLD and LC, decreased liver and increased kidney excretion of GD-EOB-DTPA resulted in an expected decrease in the liver-to-kidney enhancement ratio (LKER) in HBP images, which could be quantitatively assessed. In comparison, FLIS is based on a visual scoring assessment, so it can be defined as semi-quantitative. Therefore, this study evaluated previously unassessed relationships between PALBI grade and semi-quantitive FLIS and quantitative LKER, and between APRI and FLIS and LKER.

## 2 METHODS

This study was conducted in a tertiary-care hospital. An ethics committee approval was received for this study. (no.: 47, date: 31.10.2023). This research study adhered to the guidelines set forth in the Helsinki Declaration. Given the retrospective nature of the investigation, patient consent was not required.

In this retrospective observational study, we searched the hospital data system and identified a total of 378 patients who underwent Gd-EOB-DTPA-enhanced MRI, including the hepatobiliary phase (HBP), and had histologic and/or clinical evidence of chronic liver disease (CLD) or liver cirrhosis (LC) between January 2018 and October 2023. The images of these patients were evaluated retrospectively.

The inclusion criteria were as follows: (1) histologic and/or clinical evidence of CLD or LC. Clinical evidence of CLD was defined as progressive deterioration in liver function tests lasting more than six months [1], while histologic evidence was based on core needle biopsy specimens obtained from the liver parenchyma. Several factors that could influence the quanti-tative measurements obtained from regions of interest in the liver and kidney parenchyma were excluded. The exclusion criteria were as follows: (1) presence of malignancy, (2) liver or kidney lesions that could interfere with accurate measure-ments due to their size, (3) focal liver lesions categorized as LIRADS-3, LIRADS-4, or LIRADS-5, (4) renal dysfunction, (5) history of liver or kidney transplantation, (6) insufficient image quality for measurements, and (7) no available laboratory data (aspartate transaminase, albumin, bilirubin, prothrombin time, and platelet count) within two weeks of the MRI.

After applying the exclusion criteria, 86 patients formed the final study group. Patient selection is shown in Fig. (**1**).

### 2.1 Clinical Data Analysis

The patients' demographic, clinical, laboratory (including aspartate transaminase, albumin, and bilirubin levels, prothrombin time, and platelet number), and histopathological data were recorded from the hospital data system. APRI and PALBI scores were calculated as below:

APRI was calculated by dividing AST level (/ULN) by platelet count (10^9^ /L) and multiplying the ratio by 100 [14].

The PALBI score was calculated according to the following equation: PALBI = 2.02 × log_10_bilirubin – 0.37 × (log_10_bilirubin)^2^ – 0.04 × albumin – 3.48 × log_10_platelets + 1.01 × log_10_platelets. The PALBI grade could be divided according to the results: grade 1 (PALBI score ≤–2.53), grade 2 (–2.53< PALBI score ≤–2.09), and grade 3 (PALBI score >–2.09). The parameters were expressed in μmol/L for bilirubin, g/L for albumin, and 1,000/μL for blood platelet count [15].

### 2.2 MRI Protocol and Image Analysis

The MRI was performed using a 3-Tesla (General Electric, Milwaukee, WI) device. In all examinations, a 48-channel body coil was used. All patients underwent dynamic contrast-enhanced liver MRI consisting of axial and coronal plane single-shot fast spin echo T2-WI, axial fat compression T2WI, axial plane T1-weighted in-phase and out-of-phase images, and multiphase dynamic-enhanced axial plane liver acceleration volume acquisition (LAVA, T1-weighted, fat-suppressed, spoiled gradient recalled-echo sequences). HBP was obtained after 20 minutes from contrast injection on the axial and coronal planes. A standard dose of GD-EOB-DTPA (0.025 mmol/kg; Primovist, Bayer Healthcare) was injected intra-venously into the antecubital vein of all patients *via* the 22-G catheter.

**Fig. (2):**
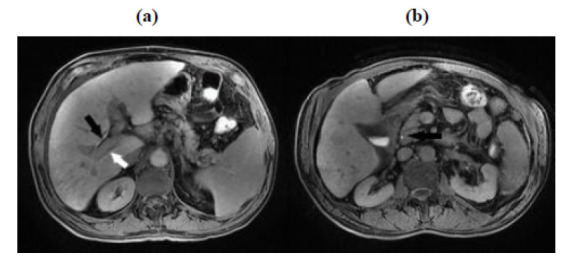
Five FLIS scores in a patient. Graft parenchymal enhancement (EnQS): SI of parenchyma relative to kidney on HBP being isointense (EnQS = 1) (**2a**). Portal vein sign (PVsQS): SI of the portal vein relative to the liver parenchyma 20 minutes after contrast application being hypointense (white arrows) (PVsQS:2) (**2a**). Contrast excretion (ExQS): the presence of contrast media in the bile ducts 20 minutes after contrast application and excretion into the common hepatic duct and the common bile duct (black arrows) can be seen (ExQS:2).

MR images of 86 patients were evaluated by a 5-year experienced board-certified radiologist. Axial and coronal planes of HBP images were used in the assessment. FLIS score was evaluated according to the features defined by Bastati *et al*. [7]. The scoring system is described in Table **1**. Scores ranging from 0 to 2 were given to each parameter. FLIS represented the sum of the three quality scores above and varied from 0 to 6 points: liver parenchymal enhancement quality score (EnQS), biliary contrast excretion quality score(ExQS), and portal vein sign quality score (PVsQS). (Fig. **2a**, **b**) represent the FLIS measurement.

For LKER, three intensity measurements were made from segments I, II, and VII using a region of interest (ROI) circle with a diameter of 1 cm, excluding focal lesions, blood vessels, and large bile ducts. These measurements were specifically selected from segments I, II, and VII to represent the caudate lobe, right liver lobe, and left liver lobe, respectively. Mean liver enhancement was calculated by the arithmetic mean of three measurements. For the assessment of renal enhancement, an ROI with a diameter of 1 cm was placed in the interpolar region of the right kidney cortex. The ROIs were placed in identical locations for all patients, both the liver and the kidney. In the absence of the right kidney, measurement was made on the left kidney. LKER was calculated by dividing the mean liver intensity to kidney intensity. The exemplary LKER measurement is demonstrated in Fig. (**3a**-**c**).

### 2.3 Statistical Analysis

All statistical analyses were performed using SPSS statistical software, version 21.0 (Armonk, NY: IBM Corp.) and MedCalc (version 15.6.1, MedCalc Software bvba, Ostend, Belgium). The normality analysis was performed using the Shapiro-Wilk and Kolmogorov-Smirnov tests. The mean, standard deviation (SD), minimum, and maximum values were obtained as descriptive statistics of continuous data, and frequency (percentage) values were taken for discrete data. For comparisons between the groups, the Kruskal-Wallis analysis of variance (ANOVA) and the Mann-Whitney U test, two of the non-parametric tests, were used. A p-value of less than 0.05 was considered significant. Spearman's rank correlation coefficient was used for determining relationships among FLIS, LKER, PALBI, and APRI. According to the correlation coefficient, the correlation was evaluated as very poor (0.00–0.25), poor (0.26–0.49), moderate (0.50–0.69), high (0.70–0.89), and very high (0.90–1.0).

## 3 RESULTS

Eighty-six patients, after applying the inclusion and exclusion criteria, were included in the study. There were 42 (48.8%) female and 44 (51.2%) male patients. The average age of the patients was 61.7±12.006 (21-88) years. Histologic evidence based on core needle biopsy specimens was present in 28 patients (32.6%), while clinical evidence of chronic liver disease (CLD) was found in 58 patients (67.4%).

The etiology was distributed as follows: hepatitis B virus in 17 (19.8%) patients, hepatitis C virus in 15 (17.4%) patients, non-alcoholic fatty liver disease in 39 (45.3%) patients, alcoholic liver in 6 (7%) patients, autoimmune hepatitis in 4 (4.7%) patients, primary biliary cirrhosis in 3 (3.5%) patients, congenital hepatic fibrosis in 1 (1.2%) patient, and primary sclerosing cholangitis in 1 (1.2%) patient. The characteristic features of the patient data group are presented in Table **2**.

Laboratory findings were as follows: mean bilirubin: 3.64±7.11 (0.19-47) mg/dL, mean albumin: 3.56±0.92 (1.3-5.5) g/dL, mean platelet count: 134.77 × 10^3^± 86.09× 10^3^ (19-413) µL, mean AST: 40.06±31.88 (12-190) U/L.

The mean APRI was 1.67±2.04 (0.13-10.55), the mean LKER was 0.99±0.36 (0.15-2.10), the mean PALBI score was -2.18±0.68 (-3.34 - -0.37), and the mean FLIS score was 4.43±1.83 (0-6).

PALBI grade was distributed in patients as follows: grade 1 in 32 (37.2%) patients, grade 2 in 25 (29.1%) patients, and grade 3 in 29 (33.7%) patients.

FLIS scores were as follows: 0 in 4 (4.7%) patients, 1 in 7 (8.1%) patients, 2 in 2 (2.3%) patients, 3 in 10 (11.6%) patients, 4 in 10 (11.6%) patients, 5 in 18 (20.9%) patients, and 6 in 35 (40.7%) patients.

The highest correlation was found between LKER and PALBI grades. Table **3** shows the correlations of APRI and PALBI grades with FLIS and LKER.

## 4 DISCUSSION

Gd-EOB-DTPA, a hepatocyte-specific contrast agent, allows to obtain both morphological and functional information about the liver. In this regard, studies have evaluated the relationship of this contrast agent with scoring systems based on clinical and laboratory findings developed for detecting liver functions. Our study evaluated the relationships of PALBI grade and APRI with semiquantitative FLIS and quantitative LKER, which has not been previously assessed. We observed a weak negative correlation between APRI and both FLIS and LKER. In contrast, the PALBI grade showed a moderate negative correlation with both FLIS and LKER. The strongest correlation was found between LKER and PALBI grades.

In CLD, liver function is crucial for predicting the stage of liver fibrosis and cirrhosis, determining the need for trans-plantation, forecasting graft survival after liver transplantation, and assessing the risk of insufficiency in patients planned for significant resection. Scoring systems include some laboratory and clinical findings to predict liver function. The most widely accepted and used ones are the CTP score, MELD score, ALBI, APRI, and PALBI. Gd-EOB-DTPA is considered a non-invasive biomarker for hepatobiliary diseases [9]. Studies in the literature have compared and evaluated the correlation between quantitative and semi-quantitative imaging (FLIS) data obtained using Gd-EOB-DTPA and commonly used liver function scoring systems based on laboratory results (ALBI) [3, 10, 12, 16-18]. Additionally, in studies comparing liver function scoring systems based on laboratory, results have shown PALBI to be more reliable than others [19-22]. Therefore, in this study, we preferred to use PALBI grade, which is more reliable and has not been used before. Also, we used APRI, which has been previously suggested as a non-invasive alternative for the exclusion and inclusion of significant liver fibrosis, since it has not been studied previously.

In a study by Luo *et al*., a weak correlation was found between FLIS and both MELD scores and ALBI grades [16]. Similarly, our study also identified a weak correlation between FLIS and APRI. Ozgul D. *et al*. demonstrated a moderate correlation between FLIS and both CTP classification and ALBI grading in patients with HCC [10]. In parallel, our study revealed a moderate correlation between FLIS and PALBI grades.

Aslan *et al*. compared FLIS with the ALBI grade and found a very strong correlation between them [3]. In Aslan *et al*.'s study, 57.2% of the patients were ALBI grade 1, whereas our study showed a more homogeneous distribution of PALBI grades. Additionally, Lee *et al*. found a strong correlation between the CTP score and the FLIS score [12]. Also, in Lee *et al*.'s study, most patients had a CTP score of A. Differences in our correlation results compared to previous studies may be attributed to variations in patient distribution. Moreover, although the FLIS score reflects liver function, the uptake of Gd-EOB-DTPA by the liver and its excretion into the biliary system are influenced by factors beyond liver function, such as liver perfusion, vascular permeability, extracellular diffusion, and blood volume [9]. Changes in these parameters among the study populations may have also influenced the results. Moreover, although scoring systems, such as ALBI grade, PALBI grade, APRI, CTP score, and MELD score, are used to predict liver function, none currently serve as the ideal gold standard, as they do not reflect all aspects of liver function. Hence, it might be necessary to develop a more ideal scoring system by combining laboratory, imaging, and clinical findings.

Although FLIS demonstrates perfect inter-reader reliability, it is a semi-quantitative method [3, 10-12]. For quantitative assessment, methods, such as relative liver enhancement (RLE), hepatic uptake index (HUI), contrast uptake index (CUI), liver-to-spleen contrast index (LSI), and LKER have been used, and it has been found that these quantitative values show moderate correlation with scoring systems [9]. Talakic *et al*. found a significant correlation between liver enzymes and LKER, similar to FLIS [23]. As LKER is a simple and objective measurement that can be obtained from HBP, we used it in our study and found that it was weakly correlated with APRI and moderately correlated with PALBI grade. Similar to the results of our study, Talakic *et al*. found a moderate correlation between cholinesterase and total bilirubin levels and LKER. In our study, the correlation of LKER with APRI and PALBI grades was similar to that of FLIS. LKER is quantitative, and its application is straight-forward with simple measurements, which may make it easier to use in daily practice than FLIS [23].

Our study involved certain limitations, primarily its retrospective nature, which may have introduced selection bias. Nonetheless, gadoxetic acid-enhanced liver MRI is routinely used as the standard diagnostic approach for patients with chronic liver disease at our institution. Secondly, our study included patients with chronic liver disease and liver cirrhosis of various etiologies, which may have masked etiology-specific associations. Subgroup analyses with larger case series may help elucidate the role of gadoxetic acid-enhanced MRI in predicting liver function for different etiologies of chronic liver disease.

A further limitation was the ±2 week window for laboratory data relative to the MRI, which could have introduced variability in liver function parameters, as they fluctuate over time. This might have influenced the correlation results with both FLIS and LKER scores. Although this timeframe was selected to minimize discrepancies, it is important to recognize that liver function can change, and a shorter or more controlled window could offer more precise assessments.

Another key limitation included the lack of a control group, which may have significantly weakened the study's ability to establish the broader diagnostic and prognostic utility of LKER beyond the scope of this study. While the correlation analysis within the patient group was valid, the absence of healthy controls prevented any claims about the ability of these scoring systems to differentiate disease states from normal liver function. This limitation hindered any conclusions about the generalizability of FLIS and LKER in establishing normalcy or their use in broader diagnostic and prognostic contexts.

Intra-reader reliability and inter-reader agreement were not evaluated. However, previous studies have stated FLIS to have excellent intra- and interobserver compatibility [3]. Also, since LKER is based on the numerical ratio of the region of interest level, it is a technique that is less affected by the different evaluations between readers.

Finally, current laboratory scoring systems have limited information on liver functions. Comparing LKER with liver biopsy data or direct functional assays, like indocyanine green, could improve the credibility and accuracy of these assess-ments, providing more comprehensive insights into liver function and enhancing the validity of the findings.

Despite all these limitations, our patient cohort reflected real-world routine practice, making the results directly applicable to clinical settings.

## CONCLUSION

Quantitative (LKER) and semi-quantitative (FLIS) tests derived from HBP showed a moderate correlation with PALBI grade and a weak correlation with APRI. This suggests that they can be used to validate rather than predict or exclude liver dysfunction. Similar performance of LKER and FLIS in determining the status of hepatic functions and the severity of the disease suggests that either may be used interchangeably. Hence, LKER is a quantitative measure with an easy appli-cation, and it is much more convenient for use in routine clinical practice compared to FLIS.

## AUTHORS’ CONTRIBUTIONS

The authors confirm their contribution to the paper as follows: concept: ACD; design: EG; supervision: EG; data collection and/or processing: ACD; literature search: ACD; critical review: EG. All authors have reviewed the results and approved the final version of the manuscript.

## Figures and Tables

**Fig. (1):**
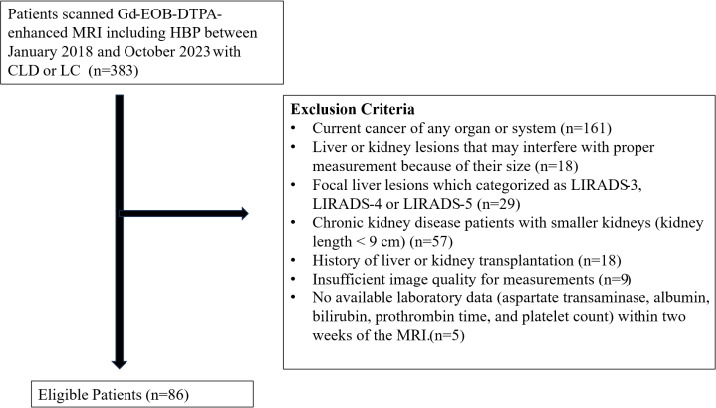
Flowchart of patient selection.

**Fig. (3):**
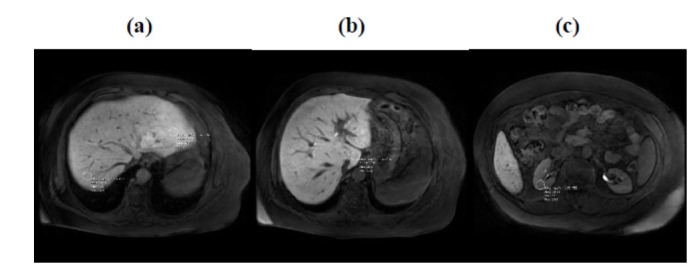
The figure illustrates that four intensity measurements were conducted on liver segments II and VII (**3a**), segment I (**3b**), and the right kidney cortex (**3c**). A region of interest (ROI) circle with a 1 cm diameter was employed for these measurements. Accurate positioning of the ROI is essential to exclude focal lesions, blood vessels, and large bile ducts that could interfere with the precision of the measurements.

**Table 1:** Definition and grading system of FLIS parameters.

**Parameter and Description**	**Score**
**Liver parenchymal enhancement (EnQS):** Signal intensity (SI) of liver tissue compared to the kidney in the hepatobiliary phase (20 minutes after contrast injection).	0 = Liver appears darker than the kidney
	1 = Signal intensity similar to the kidney
	2 = Liver appears brighter than the kidney
**Biliary contrast excretion (ExQS):** Degree of contrast excretion into the biliary tree at 20 minutes post-injection.	0 = No visible contrast in bile ducts
	1 = Contrast seen in peripheral intrahepatic bile ducts or in either right and/or left hepatic duct(s)
	2 = Contrast visible in the common hepatic duct, common bile duct, or extending into the duodenum
**Portal vein sign (PVsQS):** Signal intensity of the portal vein compared to liver parenchyma at 20 minutes post-contrast.	0 = Portal vein appears brighter than the liver
	1 = Similar intensity to the liver
	2 = Portal vein appears darker than the liver

**Table 2:** Characteristics of the patients.

**Variables**	**Patients (N=86)**
Age (years), mean (range)	61 (21-88)
Gender, n (%)	
Female	42 (48.8%)
Male	44 (51.2%)
Diagnosis, n(%)	
Clinical evidence	58 (67.4%)
Histologic evidence	28 (32.6%)
Etiology, n (%)	
Hepatitis B virus	17 (19.8%)
Hepatitis C virus	15 (17.4%)
Non-alcoholic fatty liver	39 (45.3%)
Alcoholic liver disease	6 (7%)
Primary biliary cirrhosis	3 (3.5%)
Autoimmune hepatitis	4 (4.7%)
Congenital hepatic fibrosis	1 (1.2%)
Primary sclerosing cholangitis	1 (1.2%)
PALBI grade, n (%)	
1	32 (37.2%)
2	25 (29.1%)
3	29 (33.7%)
Serum bilirubin level (mg/dL), mean (range)	3.64 (0.19-47)
Serum albumin level (g/L), mean (range)	3.56 (1.3-5.5)
INR value, mean (range)	1.72 (0.87-32)
Platelet value (10^3/uL), mean (range)	134.77 (19-413)
AST value(U/L), mean (range)	
FLIS score, n (%)	
0	4 (4.7%)
1	7 (8.1%)
2	2 (2.3%)
3	10 (11.6%)
4	10 (11.6%)
5	18 (20.9%)
6	35 (40.7%)
LKER, mean (SD)	0.9909 (±0.03893)

**Table 3:** The correlations of APRI and PALBI grade with FLIS and LKER.

-	**APRI**	**PALBI Grade**
**FLIS**	r = -0.327 p = 0.002	r = -0.495 p =0.001
**LKER**	r = -0.308 p = 0.004	r = -0.554 p = 0.0001

## Data Availability

The datasets analyzed during the current study will be available from the corresponding author [A.D] upon reasonable request.

## LIST OF ABBREVIATIONS

CLD: Chronic liver disease
LC: Liver cirrhosis
MRI: Magnetic resonance ımaging
Gd-EOB-DTPA: Gadolinium ethoxybenzyl diethylenetriamine pentaacetic acid
HBP: Hepatobiliary phase
FLIS: Functional liver ımaging score
LKER: Liver-to-kidney enhancement ratio
PALBI: Platelet-albumin-bilirubin
APRI: Aspartate aminotransferase to platelet ratio ındex
AST: Aspartate aminotransferase
ROI: Region of interest
ALBI: Albumin-bilirubin
CTP: Child-Turcotte-Pugh
MELD: Model for end-stage liver disease
NAFLD: Non-alcoholic fatty liver disease
OATP: Organic anion-transporting polypeptides
PVsQS: Portal vein sign quality score
EnQS: Enhancement quality score
ExQS: Excretion quality score
SD: Standard deviation

## STANDARDS OF REPORTING

STROBE guidelines were followed.
